# Construction and demolition waste framework of circular economy: A mini review

**DOI:** 10.1177/0734242X231190804

**Published:** 2023-08-31

**Authors:** Iliana Papamichael, Irene Voukkali, Pantelitsa Loizia, Antonis A Zorpas

**Affiliations:** Faculty of Pure and Applied Sciences, Laboratory of Chemical Engineering and Engineering Sustainability, Open University of Cyprus, Latsia, Nicosia, Cyprus

**Keywords:** Construction and demolition, waste, circular economy, models, material recovery, key performance indicators

## Abstract

As the demand for materials continues to increase and building lifespans shorten, the construction industry faces mounting pressure to reduce its material and environmental impacts. Mismanagement of construction and demolition waste (CDW) can have severe environmental consequences. To address this, material recovery and circular economy approaches offer significant potential for reducing construction waste through the sustainable use of resources. Existing circular economy and material recovery models that prioritize recycling and reuse efforts demonstrate a sustained commitment to supporting circular practices in the construction and demolition sector. The goal is to minimize waste production, which poses environmental challenges such as raw material shortages and sustainability concerns. Using Preferred Reporting Items for Systematic Reviews and Meta-Analyses statement for recruiting relevant literature, this mini review aims to identify the obstacles to implementing circular economy practices in the construction industry, while exploring opportunities for material recovery and circularity. The ultimate aim is to facilitate a fair and smooth transition towards sustainable development, while addressing environmental, social and economic barriers. A more sustainable and circular approach to building construction and management can be attained by considering all the aspects of the CDW cycle, resulting in significant benefits for the environment and society as a whole.

## Introduction

The most critical challenge that humanity will face in the near future is global warming, and immediate action is required to reduce the use of fossil fuels and raw material that contribute to greenhouse gas emissions. Delaying this necessary action is no longer an option (D’Adamo et al., 2023). The construction industry continues to face intense pressure to reduce its environmental and material impacts while the increasing material demand and decreasing building lifespans have prompted the research community as well as governments to take a closer and more attentive look at the situation (Arora et al., 2021). According to Yang et al. (2022), 25% of natural resources are consumed by construction operations while generating one-fifth of municipal solid waste (MSW). In 2016, MSW landfill rate in the EU was almost 23%, which was too high in relation to the objectives of less than 10% by 2035, according to the European Green Deal. Simultaneously, the incineration rate decreased between 2001 and2014 to 32%, but was still considered too high, accounting for 26% of MSW (Chioatto and Sospiro, 2023). Concerning construction and demolition waste (CDW), in the United States (US) alone, CDW amounted for 534 million tonnes (67%) of solid waste produced, whereas for the European Union (EU), 36% (924 million tonnes) was accumulated in 2016 (Chen et al., 2021).

CDW comprises of numerous materials, including concrete, bricks, wood, glass, metals and plastic. It includes waste from the construction and demolition of buildings and infrastructure, as well as the planning and maintenance of roads (European Commission, n.d.). According to Statista (2022c), in 2020, the amount of mineral waste from CDW (i.e. asbestos, natural occurring minerals, soils treated) in Spain amounted to 38.31 million metric tonnes for a 10-year period (2010–2020). At the same time, the volume of demolished buildings in Germany decreased by 82,300 m^3^ from 2019 to 2020, whereas in 2020 alone, 32,200 m^3^ of residential buildings were demolished (Statista, 2022b). Simultaneously, in 2021, Italy produced 402.9 thousand tonnes of CDW, fluctuating between years 2019 and 2020 from 390.3 to 429 thousand tonnes, respectively (Statista, 2022c). Apart from the EU, in 2020, the volume of CDW in Kuwait was 13.7 million metric tonnes (Statista, 2022d). Recovery rate for CDW in the EU was 88% in 2018, whereas Member States set a recovery target of 70% by 2020 according to the Waste Framework Directive (WFD) (European Commission, 2008; Statista, 2022a).This recovery target includes all forms of recycling and other recovery operations, such as backfilling. However, some EU member states have relied heavily on backfilling or low-grade recovery methods, which make up a significant portion of their overall recovery rates, despite having already achieved the target (Statista, 2023). The three bottom countries are Cyprus, Slovakia and Bulgaria with 64, 51 and 24% recovery rate, respectively.

According to Wu et al. (2019), the global amount of CDW generated annually is over 10 billion tonnes, with the US and the EU producing about 700 million tonnes and more than 800 million tonnes, respectively. China is responsible for producing about 2.3 billion tonnes of CDW each year, accounting for around 40% of total solid waste generated, as a result of rapid urbanization and extensive urban renewal projects (Chen et al., 2021). The worldwide impact of CDW on the environment has become a major concern. Currently, in many regions of the world, landfill is the primary method of CDW treatment, and approximately 35% of CDW is estimated to be landfilled globally (Kabirifar et al., 2020).

The construction and demolition industry consumers 40% of raw stone, gravel and sand usage, as well as almost 25% of wood each year. Simultaneously, it accounts for 23% of air pollution (greenhouse gasses and particulates matter emissions) amounting to 50% of climate change, 40% drinking water pollution and 50% of landfilling (Kabirifar et al., 2020). According to Sinoh et al. (2023), concrete production amounts of more than 30 billion tonnes on a yearly basis, amounting for 8% of global carbon dioxide emissions, whereas its aggressive consumption leads to the unsustainable exploitation of natural aggregates. Due to the large volume and size of waste from construction, CDW take up a lot of space in landfills, leading to lack of space for other waste (thus the creation of new landfilling sites), increase in air, water and soil emissions (carbon dioxide, leachate leading to increase in soil and water toxicity, etc.). At the same time, the continues construction activities of the industry cause a vast habitat destruction and ecosystems, leading to a severe loss of biodiversity and ecosystem services (Kabirifar et al., 2020).

The contribution of CDW to environmental pollution is not limited only to the production and accumulation of waste. According to Chen et al. (2021), CDW generate rapidly due to urbanization and railway transportation occupying land resources and causing the depletion of landfills (Colorado et al., 2022). At the same time, the large amount of CDW is attributed to the fact that CDW have much higher volume than other types of waste. At the same time, illegal dumping sites along with waste leachate pose extreme risks as CDW contains various pollutants. Such compounds include heavy metals, organics and inorganics like hexabromocyclododecane and polybrominated diphenyl ethers, chlorides, sulphides, organic carbon, oil, fuel, solvents bringing upon an increasing risk to surrounding water, groundwater and soils causing environmental and landscape degradation (Tafesse et al., 2022). Additionally, CDW along with other construction operations lead to land space, air, noise and water pollution. Furthermore, resource depletion (35% of resources used) is a major concern regarding demolition activities of the industry, leading to energy consumption (40% of total energy consumed) for the manufacturing and transportation of new materials while simultaneously accounting for 12% of the world’s potable water and 40% of global carbon dioxide emissions (Bertino et al., 2021; Chen et al., 2021; Tafesse et al., 2022). The sector also generates approximately one-third of all waste destined for landfills (due to heavy construction activities and bigger size of waste) and is associated with different stages of a building’s lifecycle, including the manufacturing of construction products, building construction, use, renovation and the management of building waste. As CDW consists of wide range of components and different (non-)renewable materials, such as concrete, bricks, gypsum, wood, glass, metals, plastic, solvents and excavated soil, many could be recycled (Bertino et al., 2021). In 2013, in the United Kingdom, 44% (58 million tonnes) of landfill waste was produced due to construction activities, whereas the rest included household, industrial, commercial, agricultural and mining waste. At the same time, more than 50% of CDW in the country is directly landfilled (Ghaffar et al., 2020).

Due to the environmental burden of CDW, social factors associated with their effect on delaying the transition towards more sustainable cities and communities (sustainable development goal (SDG) 11) constitute a primary area impacted. CDW can lead to safety hazards for workers and nearby communities, causing accidents or injuries while unsightly and unsanitary conditions, causing a decline in property values and negatively impacting tourism and local economies as well as the quality of life of close by residents (Wu et al., 2023). According to Tafesse et al. (2022), among the most significant social effects of CDW are health risks associated with diseases due to CDW air pollutants, traffic congestion, drainage blocking along with waste transported by rainwater. Their research indicated that the potential for human health and social wellbeing disturbance form CDW arise since it is typically discarded near residential areas and pollutes the environment significantly. Regarding economic burdens, the construction sector constitutes 12% of the global gross domestic product while it consumes a large amount of raw materials, electricity and equipment, chemicals and other services (Bertino et al., 2021).

In this regard, the EU has taken several steps to tackle CDW implications on sustainability pillars. WFD (2008/98/EC) is a legislative framework established by the EU to promote a sustainable approach to waste management. This directive sets out various measures to ensure the proper handling and disposal of waste, including CDW. The directive emphasizes the importance of the ‘waste hierarchy’ approach, which prioritizes waste prevention, reuse and recycling over disposal. By adopting this approach, the construction industry can significantly reduce the amount of waste generated and promote a more sustainable approach to construction. Overall, the WFD plays a crucial role in regulating CDW management practices in the EU and promoting sustainable waste management practices in the construction industry. The directive targeted an increase of 70% by weight of the preparation of CFW for reuse, recycle and material recovery for non-hazardous waste by 2020 and the promotion of selective demolition with safe handling of hazardous materials and compounds. This would facilitate the reuse and high-quality recycling of selected materials towards the reduction of waste generation (Official Journal of the European Union, 2008).

Furthermore, the European Commission introduced a set of non-binding protocol and guidelines for industry practitioners, public authorities, quality certificate bodies and clients for the management of CDW (European Commission, 2018). The protocol fits with the ‘Strategy for the sustainable competitiveness of the construction sector and its enterprises’ and the communication on ‘Resource Efficiency Opportunities in the Building sector’, with the aim of increasing momentum on CDW management and material recovery (European Commission, 2012, 2014). The strategies aim at improving waste identification, separation and collection, optimize waste logistics and processing and provide quality management in an appropriate policy framework.

At the same time, international bodies, like the International Organization for Standardization (ISO), have provided guidelines for organizations to improve their environmental performance by establishing management systems and implementing best practices for environmental management. ISO 14001 is the international standard that outlines the requirements for an environmental management system (EMS) for organizations (Beuron et al., 2020). This standard provides guidelines for identifying and managing the environmental impacts of an organization’s activities, products or services, including CDW. ISO 14001 can be used by construction companies and other organizations to establish an effective EMS for the management of construction waste. The standard requires organizations to assess their environmental impacts, including those related to waste generation, and implement measures to reduce and manage these impacts. ISO 14001 also provides guidance on the establishment of waste management plans, including the identification of waste streams, waste reduction and prevention measures and the implementation of waste recycling and disposal strategies. The standard emphasizes the importance of continuous improvement, monitoring and evaluation of the effectiveness of the waste management system. In addition to ISO 14001, there are other standards related to construction waste, including ISO 9001 for quality management systems, ISO 45001 for occupational health and safety management systems and ISO 50001 for energy management systems. These standards can be used in conjunction with ISO 14001 to establish a comprehensive management system for sustainable construction practices.

Linear economic models at hand are based on a ‘take, make, dispose’ mindset implemented from raw materials extraction, production (on site), construction and disposal (Benachio et al., 2020; Yang et al., 2022). Circular economy depicts an economy system based on business models, which replace linear production and disposal practices with circular ones (Oliveira et al., 2021). It replaces the end-of-life concept with reduce, reuse, recycling and other alternative waste management practises and strives in keeping products and materials ‘in the loop’. This is accomplished through effective and sensible re-use tactics, which reduces the consumption of virgin resources and negative environmental implications (Ghaffar et al., 2020). As a novel economic and production paradigm, circular economy entails a mindset shift that views waste as potentially usable resources rather than a burden to manage and dispose of in landfills, as it did in the previous linear economy. Circular economy solutions find the best ultimate treatment options for waste materials, reducing waste creation at the source and minimizing waste volume through enhanced efficiency, recycling and suitable design. One of the most important aspects of circular economy in the context of CDW is the recycling and/or recovery of CDW, which is practiced in several European nations. However, due to a variety of causes, a huge volume of potentially reusable/recyclable goods are legally or illegally disposed (European Environment Agency, 2020; Ghisellini et al., 2018).

In this regard, on March 2020, the European Commission adopted the new circular economy action plan (European Commission, 2021; The World Bank, 2022). As a key component of the European Green Deal, the new strategy for long-term growth. The EU’s move to a circular economy will relieve pressure on natural resources while also fostering long-term development and job creation. It is also required in order to meet the EU’s 2050 climate neutrality of the European Green Deal and reverse biodiversity loss. The ‘New Circular Economy Action Plan – For a cleaner and more competitive Europe’ is considered a key strategy to achieve the aforementioned objectives. It recognizes various industries, including construction and demolition, the textile industry, etc. as crucial sectors in transitioning to a circular economy due to their potential for resource conservation. The plan outlines measures that need to be implemented, such as harmonizing waste collection practices across the EU and promoting sorting and recycling activities. At the same time, the ‘Strategy on Chemicals for Sustainability – Towards a Toxic Free Environment’ addresses the safe recycling of materials while focusing on the early adoption of safe and sustainable chemicals throughout a product’s life cycle, emphasizing the importance of non-toxic materials in creating a sustainable and circular economic and production system (De Ponte et al., 2023).

Following the implementation of the circular economy package, the EU has made revisions to its significant waste management directives. These directives govern various aspects of waste management, encompassing measures for prevention and treatment such as landfilling, incineration (for disposal and energy recovery), recycling, composting and digestion. In this context, the newly introduced Directive 2018/851 has strengthened the objectives concerning MSW recycling and reuse, aiming for a recycling and reuse rate of 55% by 2025, 60% by 2030 and 65% by 2035. Additionally, Directive 2018/850 has redefined the objective for reducing MSW landfilled to below 10% of the total MSW generated by the year 2035 (Chioatto et al. 2023).

Furthermore, in the ‘Roadmap to a Resource-Efficient Europe’ of 2020, optimization of waste management is one of the main pillars of the strategy, whereas reduction, reusing and recycling waste constitute few of its main goals (European Commission, 2011). EU policies (i.e. resource efficiency opportunities in the building sector, strategy for the sustainable competitiveness of the construction sector and its enterprises, construction and demolition guidelines and protocol) seem to recognize such implications of CDW on sustainability pillars (European Commission 2012, 2014, 2018). Therefore, immediate mitigation of those effects are a priority for greenhouse gases (GHGs) emissions reduction, climate change mitigation and resource depletion, by shifting the focus on the adoption of circular economy. Simultaneously, taking into account the SDGs of the United Nations (UN), such policies, strategies and directives will aid to meet the standards of the agenda by 2030. Specifically, the EU strives to promote the development of policies to support productive activities and job creation through CDW management (SDG 8 – decent work and economic growth) while reducing the per capita environmental impact of cities towards sustainable communities (SDG 11 – sustainable cities and communities). Simultaneously, the sustainable management of raw materials and material recovery will assist with mitigating overconsumption of natural resources (SDG 12 – responsible consumption and production) and aid with overall mitigation of environmental degradation (SDG 13 – climate change) (UN, 2015). SDG 12 specifically, incorporates sustainable consumption and production practices, which aim to reduce waste generation by adopting preventive measures, promoting reuse, recycling, and recovery of waste. To achieve this goal, new models of consumption and production are being developed. Additionally, the notion of waste as a valuable resource is being embraced, and as a result, landfilling is viewed as a last resort in the sustainable management of waste (Appolloni et al., 2021).

The current mini-review focuses on circular economy and material recovery opportunities for CDW as well as barriers that may arise for circular economy implementation and how to overcome them. Furthermore, tools and methods (such as key performance indicators (KPIs)) used to measure the level of CDW circularity are discussed.

## Methodology

**Figure 1.**
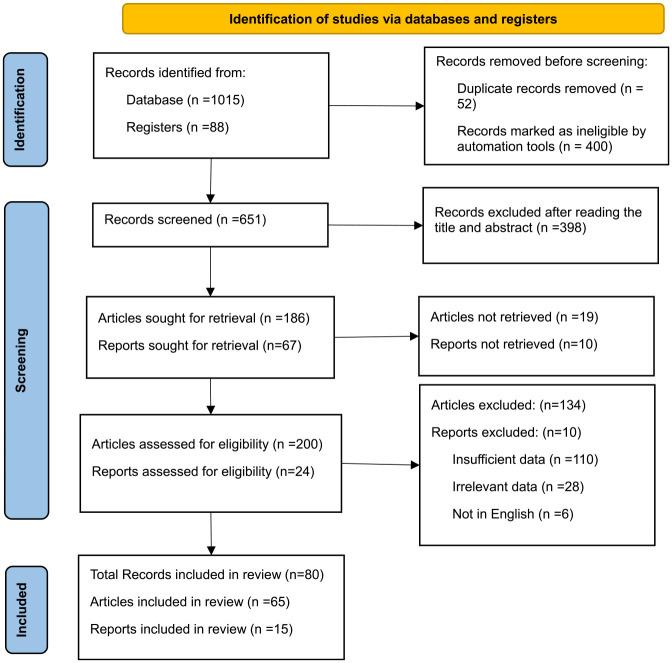
Prisma statement of the current review.

Preferred Reporting Items for Systematic Reviews and Meta-Analyses (www.prisma-statement.org) statement (Figure 1) was used for the strategic exploration and data synthesis of the principal findings with which this research was developed, by counting eligibility and exclusion criteria and relating sources through 27 routes and incorporating well-defined stages of systematic reviewing (Chatziparaskeva et al., 2022; Moher et al., 2015).

From the 1103 records identified for the paper (1015 articles from Scopus database and 88 reports from other registers, 51 were selected pertaining to CDW, material recovery and the circular economy. The inclusion criteria were: (i) articles on CDW (ii) studies presenting the impacts of CDW on sustainability pillars, (iii) review articles, (iv) articles published in English, (v) articles published between 2019 and 2023 and (vi) records concerning CDW statistics. The exclusion criteria included (i) duplicate papers and publications, (ii) papers focusing on CDW in areas other than waste management and sustainability, (iii) technical reports and (iv) everything not included in the inclusion criteria. Using the Scopus database, a Boolean keyword search for ‘construction and demolition waste’ AND ‘material recovery’ AND/OR ‘circular economy’ was conducted. In order to minimize errors, each paper’s title and abstract were screened for decision-making purposes prior to review. To minimize bias, the review team debated whether a publication should be considered in the event of disagreement. Scopus references were cross-referenced in Mendeley before being downloaded for full-text evaluation.

The authors extracted data from 65 articles and 15 reports, 80 records in total. The data were used to describe the current status of CDW as well as circularity opportunities, including but not limited to material recovery, in order to shed light on the advantages of developing an appropriate waste management strategy for the construction industry.

## Results and discussion

CDW classification varies according to its source or its nature. According to the European Waste Catalogue of SEPA (2015), Construction wastes are characterized as ‘all wastes that originate from construction activities, such as rubble, asbestos and plasterboard’. These also are classified as mixed CDW, wood, bricks, concrete, soil and stones, tiles and ceramics. Other classifications, according to Purchase et al. (2022), when it comes to the nature of the CDW, it is categorized into physical waste (i.e. residue) ad non-physical waste (i.e. time and cost consumption). Another classification includes manmade CDW (i.e. design, handling, operation, residue, etc.) or from natural sources (i.e. earthquakes, floods, natural weather phenomena, etc.). CDW is produced not only from planned construction, renovation and demolition activities but also from unexpected catastrophic events. For instance, the 2016 earthquake in Central Italy affected 140 municipalities, devastated an area of almost 8000 km^2^ and generated significant amounts of rubble. Proper management of CDW in this context is critical, as it can impact the reconstruction process of buildings and undermine overall progress (Volpintesta et al., 2023). Specific to earthquakes, the impact of surface rupture on civil structures has a significant influence on their earthquake response. Surface ruptures, which are often triggered by powerful earthquakes, are a crucial aspect to consider in any construction project. Among all natural disasters, earthquakes and tsunamis pose a particularly high risk due to their sudden and unpredictable nature, creating a double disaster scenario (Fitwi, 2023).

**Figure 2.**
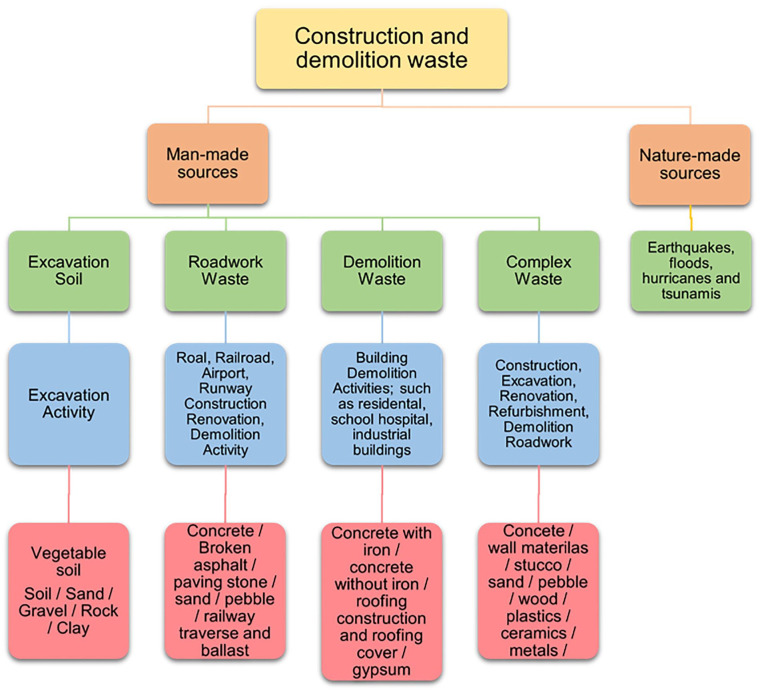
Categorizing CDW (Chen et al., 2021). CDW: construction and demolition waste.

The manmade category could be further subdivided into the following subcategories: (i) waste from excavation soil (soil, sand, rock, clay, etc.); (ii) roadwork waste (concrete, broken asphalt, paving stone, sand, etc.); (iii) demolition waste (concrete with iron, concrete without iron, roofing, etc.) and (iv) complex waste (concrete; wall materials, pebble, wood, plastics, etc.) (Arslan et al., 2012; Chen et al., 2021; Nawaz et al., 2023) (Figure 2).

The main causes of CDW production on site arise from site conditions and lack of efficiency of waste handling due to inadequate infrastructure (i.e. bins), lack of knowledge on waste collection and CDW implications (i.e. education, training of staff) but also lack of national and regional legislation on CDW disposal (Eliades et al., 2022; Loizia et al., 2021; Zorpas et al., 2021). Through the designing of new strategies, site culture is a very important pillar for the adequate transition towards circularity. The implementation of strict waste audits and monitoring of CDW production through indicators (i.e. waste index) will be deemed very important in the reduction of on-site waste. This can be achieved through the conduction of seminars and meetings for educating employees and employers on the importance of CDW reduction on site (Purchase et al., 2022).

### Material recovery and circularity of CDW

In recent years, as awareness of sustainability and resource management has increased, several nations have begun to investigate innovative approaches to minimize the use of finite resources that are currently overutilized, poorly managed or fast exhausted. In this context, the adoption of circular economy has emerged as a promising strategy to reduce the negative environmental impact of CDW (Purchase et al., 2022). High rates of recovery and recycling in the construction sector need closed material cycles and unmixed material fractions. The increasing diversity of CDW makes it more difficult to distinguish between the many components. Manual sorting exposes the executing crew to several risks and depends exclusively on visually visible differences for separation (Klewe et al., 2022). In addition, to properly apply circular economy in a construction company, numerous dimensions, such as social, governmental, economic, behavioural, technological and environmental factors, must be clarified in detail.

In today’s society, it is clear to see that the ‘mine-build-discard’ mindset concerning CDW is far from sustainable (Purchase et al., 2022). Existing waste management strategies usually consist of the 3R concept of reduce (avoid the production of waste), reuse (utilization of waste in a viable manner) and recycle (recycling of CDW to add value to material recycling). Such approaches have the capacity of reduction landfilled CDW while assisting not only to the reduction of consumption of natural resources but also to the conservation of the economy. Still the 3R must not be treated as an individual concept since the recycling of CDW does not entail the reduction of waste at source. Still, construction practitioners should take into account the integration of measure of the minimization and recycling of waste on site (Purchase et al., 2022).

A typical building has a projected structural and useful lifespan of 50–100 years, but after less than 30 years it is frequently seen to have lost value due to loss of attractiveness and functionality and is therefore demolished and replaced by a new one. However, if a building’s value is preserved and it is utilized more and for a longer period of time, its materials may be kept out of landfills and incinerators, and the demand for new materials can be greatly decreased. Simply increasing the lifespan of existing buildings might cut annual greenhouse gas emissions by 1.3 billion tonnes of CO_2_ eq. by 2050.

Due to design decisions and overspecification, construction projects frequently consume more resources than necessary. For instance, it is frequently possible to produce the same structural strength using 55% of the prescribed cement. The construction and refurbishment of buildings account for 11% of total greenhouse gas emissions in urban areas, with the majority of these emissions being from the manufacturing of cement, steel, aluminium and plastic. Therefore, the push to upgrade buildings towards energy efficiency and renewable energy usage must be accompanied by efforts to reduce the emissions generated during the production of construction materials, known as embodied emissions, as well as those that result from how CDW are disposed of (Bergonzoni et al., 2023; Purchase et al., 2022).

Concrete is one of the most valuable materials of CDW as it comprises of many finite materials, which contribute to the production of CO_2_. Various materials (i.e. glass, polyethylene terephthalate (PET), ceramics, clay bricks, tires, concrete waste, agricultural waste, silica flume, fly ash, etc.) can be used in concrete production to reduce the disposal of large volumes of CDW through aggregates replacement (Bergonzoni et al., 2023; Purchase et al., 2022).

When using recycled materials in concrete, such as tires, PET, ceramics and demolition aggregates, it is important to consider the amount of water required for mixing. Recycled materials may have different porosity, density and absorption characteristics compared to traditional aggregates, which can affect the amount of water needed to achieve the desired consistency and strength of the concrete (Dawood et al., 2021; Umasabor and Daniel, 2020).

Moreover, it is crucial to provide detailed information on the physical and mechanical properties, behaviour and durability of each recycled material used in concrete. For instance, recycled ceramic aggregates have a lower water absorption capacity than natural aggregates, which can improve the durability of the concrete. On the other hand, using PET plastic in concrete can result in reduced strength and stiffness, but it can also enhance the concrete’s ductility and impact resistance (Dawood et al., 2021; Umasabor and Daniel, 2020).

Specifically, the maximization of reuse and recycling of CDW results in a decrease of landfilled waste therefore prolonging the lifespan of existing landfill sites and aversion of chemicals from CDW onto landfill leachate. Consequently, the toxicity onto groundwater can be somewhat averted contributing to the reduction of toxic substances causing severe health issues escape, whereas transportation requirements for large volume CDW can be diminished. Therefore, the diminishment of landfill use may lead to a loss of employment, which will be counterbalanced by the creation of new job opportunities. At the same time, the long-term reduction of landfilled CDW may lead to a decrease in land demand for new landfill sites.

Recycled aggregates may have several applications and are categorized into low-quality recycled aggregates (i.e. environmental filling, rehabilitation of depleted quarries and landfill sites), medium-quality recycled aggregates (i.e. Airport, road, harbour construction, etc.) and high-quality recycled aggregates (i.e. concrete production and road construction) (Ghisellini et al., 2018; Paz et al., 2023). For instance, different forms of concrete (i.e. conventional concrete, geopolymer concrete, etc.) rely on natural aggregates as their main component, comprising 75–80% of the total volume of concrete. Due to the high demand for aggregates in concrete production, the current over-reliance on Not applicable (NAs) has caused ecological harm and depletion of natural sand and gravel resources in certain regions. In addition, inappropriate disposal of construction waste has also contributed to environmental issues. Therefore, the development of green concrete is necessary to address these concerns. Recycled aggregate concrete (RAC) is a promising material that replaces NAs partially or entirely with recycled aggregates. It can reduce energy consumption and mitigate the accumulation of construction waste. Studies have shown that the quality of recycled aggregates, which directly affects the mechanical properties and durability of RAC, is influenced by various factors. Recycled aggregates can be further classified into ‘mortar-covered aggregates’ and ‘mortar-attached aggregates’, with the former demonstrating superior water absorption compared to the latter (Zhang et al., 2023).

The addition of aggregate waste in concrete has different benefits to its properties. For instance, the addition of glass in different forms (i.e. coarse glass aggregate, fine glass aggregate, glass powder) created pozzolanic reaction, which in its essence reduces GHGs emissions (CO_2_ and nitrous oxides) from concrete. Furthermore, due to its high thermal conductivity, glass aggregates can be used on building for thermal stability (Purchase et al., 2022). Harrison et al. (2020) found promising results for aggregate replacement of waste glass (20%) in cement materials arising from its pozzolanic reactions promoting the mechanical properties. Similarly, through the research of Zheng et al. (2021) for modified recycle concrete, the authors managed to improve the durability of recycled concrete through the addition of nano-SiO_2_ and basalt fibre while investigating mechanical properties, interface structure, carbonization freeze, salt erosion and high temperature resistance for nano-SiO_2_ concrete, basalt fibre concrete and fibre nano-recycle concrete. The authors depicted that the addition of these compounds can improve certain characteristics of the concrete material (i.e. interface structure and durability).

Apart from glass, PET can be used in concrete with huge environmental benefits. As PET waste percentage increases, the workability of concrete decreases, with slumps decreasing by 12.5 and 62% for 5 and 20% PET replacements, respectively. The absorption rate increases with increasing PET waste content, with 20% replacement resulting in a 55.4% greater absorption rate than reference specimens, whereas compression, splitting tensile, and flexural strengths increase with PET waste content up to 12.5%, with optimum replacement at 7.5% resulting in strength increases of 43.64, 26.9, and 30.2% compared to the reference mix. At the same time, ductility increases with increasing PET particle ratio in concrete, as concluded from the failure mode of different specimens. The use of PET plastic waste in concrete eliminates non-degradable, harmful waste and prevents future environmental pollution (Dawood et al., 2021).

Due to its lightweight properties, PET can be used in light weight concrete used to reduce dead weight of structure while at the same time maintaining high quality, maintain corrosion resistance, density, elasticity and tensile strength (Purchase et al., 2022). Bachtiar et al. (2020) investigated the influence of recycled PET (R-PET) artificial aggregate as a substitute for coarse aggregate on the compressive and flexural strength, as well as the volume weight of the concrete. PET plastic waste was recycled by heating it to a temperature of around 300°C. Slump, bleeding and segregation tests were performed on new concrete mixes while compressive and flexural strength tests were performed in accordance with ASTM 39/C39M-99 and ASTM C293-79 standards. The findings indicated that using PET artificial aggregate might increase the workability of the concrete mixture. The influence of PET artificial aggregate as a coarse aggregate substitute on the compressive and flexural strength of concrete is thought to be substantial. The reductions in concrete volume weight were 8.45, 17.71, 25.07 and 34.60% of the weight of the conventional concrete volume (2335.4 kg/m^3^).

Additionally, marbles, ceramic and tiles can also be used to improve concrete properties as aggregates. Marble, ceramic and tiles are examples of CDW materials that can be recycled and reused in the production of concrete. These waste materials are commonly found in CDW and can be processed into fine particles or aggregates to replace a portion of the natural aggregates used in concrete production. The use of recycled marble, ceramic and tiles in concrete can provide several benefits, including improved mechanical and durability properties of the concrete. For example, the addition of finely ground ceramic waste to concrete can improve its compressive strength, flexural strength and abrasion resistance, while reducing its permeability and water absorption. Similarly, the use of marble waste in concrete can improve its compressive strength and reduce its water absorption. In addition to improving concrete properties, the use of recycled marble, ceramic and tiles in concrete can also contribute to sustainable construction practices by reducing waste generation and conserving natural resources. By diverting these waste materials from landfill and incorporating them into concrete production, the industry can reduce the need for virgin materials, thereby conserving natural resources and reducing environmental impacts associated with material production (Kumar Goyal et al., 2022).

The use of 10–20% of coarse grains ceramics increases concrete compressive strength and decreases specific weight without affecting water absorption significantly. Furthermore, tiles and ceramics have low specific weight and good pozzolanic properties, but still their addition and aggregates should be checked beforehand as their characteristics can vary according to their manufacturing process. At the same time, waste tires have limited recycling capabilities yet when used for concrete aggregates, they give the additional benefit of reducing concrete stiffness for fire protection and increase flexural strength (Purchase et al., 2022). According to Meena et al. (2022), the fresh density and hardened density of concrete containing ceramic waste aggregates decrease linearly as the ceramic concentration increases. The hardened qualities of concrete often decrease when the ceramic component is increased. However, ceramic concrete has a slightly higher compressive, flexural, split tensile strength and modulus of elasticity while absorbing more water than conventional concrete. At the same time, the depth of chloride-ion penetration in concrete decreases as the ceramic content increases, whereas electrical resistance of concrete increases as the amount of ceramic increases. Lastly, the durability of concrete normally increases as the ceramic percentage increases.

Furthermore, agricultural waste (i.e. coconut, bamboo, straw, wheat straw, etc.) can also be used in concrete. Agricultural waste refers to waste from agricultural production, product process, livestock, breeding and rural residents. They account for more than 30% of the world’s agricultural output, whereas in most cases they are burnt or landfilled (Gao, 2021). For concrete application, agricultural waste use includes natural plant fibres, sisal fibre, date palm fibres, agricultural waste ash, olive waste ash, banana leaf ash and others. According to Purchase et al. (2022), almond and coconut shells can be employed in this regard, as almond shells usage as a coarse aggregate generates average slump, higher air content and reduced air density when compared to regular concrete. Also, coconut shells create a lightweight, high-quality concrete. Similarly, Gao (2021) studied the application of agricultural waste on concrete, depicting that it can significantly minimize the environmental impact of typical concrete manufacturing. Throughout the author’s results, the addition of agricultural waste seemed to either increase or maintain mechanical properties of concrete. For instance, the addition of 5–15% of date palm fibre improved composite materials’ thermal and mechanical properties, whereas adding 2.5% by weight of cement of hibiscus fibre increases the impact resistance of cement mortar.

Interestingly enough, concrete itself can be aggregated and reused in the construction industry. The economic viability of recycled concrete as aggregate suggests that its utilization as construction material reuse produces a net benefit of approximately 31 million US dollars per year while diminishing resource depletion and energy use. Still, such incentives have the particularity of lack of availability of recycled concrete while the inconsistencies in quantities (due to seasonality or price fluctuations) does not allow for adequate economic budgeting and revenue stream (Purchase et al., 2022).

### Circular economy model for CDW

According to López Ruiz et al. (2020), an integral circular economy model for CDW includes the five stages of their production from preconstruction, construction and renovation, collection and distribution and end-of life management. Nevertheless, the conceptualization of circular economy models in the construction industry includes seven main stages from extraction and use of raw materials, material input, design, construction and production, distribution, collection and recycling. According to, Ellen MacArthur Foundation (n.d), there are three primary circular economy initiatives that target emissions from the built environment directly: (i) maximization of use of existing buildings by sharing and reusing them, (ii) design of new structures for adaptable materials and (iii) reuse and recycle of CDW to prevent disposal in landfills or incinerators. Together, these methods may cut global CO_2_ emissions from the construction and destruction of buildings by 2.1 billion tonnes by 2050, making them essential to achieving net-zero emissions.

The main contributor to circular economy from the construction sector is the recovery, reuse and recycling of CDW. The recovery, reuse and recycling of CDW are essential components of the circular economy in the construction sector. By extending the life cycle of materials through recycling and reuse, the industry can reduce waste generation, conserve natural resources and reduce greenhouse gas emissions associated with material production. Furthermore, the use of recycled materials in construction can reduce the environmental impacts associated with material production, such as energy consumption, water use and carbon emissions (Angrisano and Fabbrocino, 2023; Norouzi et al., 2021). Circular economy principles in the construction sector also extend to the design and construction phases of projects. For example, designing buildings for deconstruction and reuse can facilitate the recovery of materials at the end of their useful life, reducing waste generation and contributing to circularity. The use of prefabrication and modular construction techniques can also facilitate the recovery and reuse of materials, as components can be easily disassembled and reused in other projects (Iacovidou et al., 2021).

Regarding this, the involvement of circular economy and material recovery has a great potential for the reduction of landfilled CDW through the utilization of such materials in a sustainable manner. Therefore, adding to the 3R of circular economy is recovery regarding raw materials. Through such an approach, the consumption of raw materials slows down bringing upon environmental (i.e. reduction of GHGs emissions), social (i.e. pleasant environment) and economic (i.e. reduction of cost) benefits (Purchase et al., 2022). The comprising materials of CDW are considered high value materials with the potential to be recycled for the construction of concrete. According to Purchase et al. (2022), CDW compositional analysis in New Zealand included concrete, plastic, wood, iron and metals, miscellaneous, glass and hazardous materials and organic waste in a percentage of 25, 19, 38, 6, 5, 2 and 2%, respectively.

As part of the transition from a linear economy to a circular economy, which has recently raised the interest of researchers, policymakers, governments and industries all over the world, the current construction practices need to be re-examined, in order to create insight on their strengths, weaknesses and barriers in their design for the transition towards circularity in the construction sector. This requires taking into account new and improved methods and services, minimizing the environmental impacts and allowing for the reuse of buildings’ components and materials in order to avoid waste and reduce costs (Bertino et al., 2021).

The implementation of circular economy comes with a variety of barriers and shortcomings, which need to be evaluated to create a safe and innovative environment in the construction industry (Liu et al., 2021; Scarpellini et al., 2019). Some of the existing barriers to this regard include: (i) lack of creation, establishment or implementation of policies and governance on behalf of policymakers and stakeholders; (ii) lack of monitoring of quality conditions for high-quality products; (iii) lack of information within the industry regarding the importance of circularity, opportunities of circular economy and CDW; (iv) associated cost and capital of recycling, reusing and recovering of CDW; (v) cultural perception of the value of CDW within (i.e. employers, employees, etc.) and outside (i.e. citizens, governmental bodies, etc.) the industry; (vi) lack of technological knowhow regarding circularity of the industry; (vii) lack of education and environmental awareness on CDW implications for different players (i.e. industrial bodies, governance, citizens, etc.) and (viii) lack of standards regarding the recycled and recovered materials (Purchase et al., 2022).

**Figure 3.**
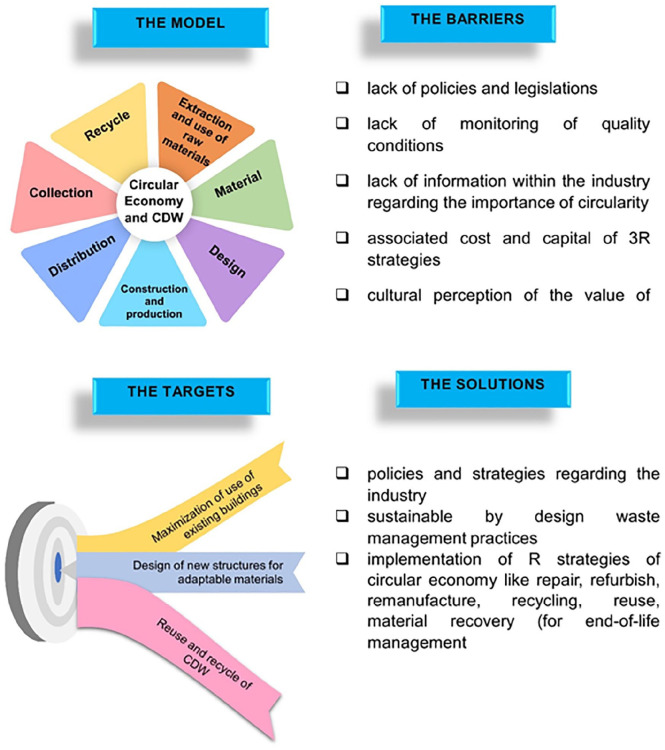
Circular economy model for construction and demolition waste.

To tackle such barriers, according to many researchers (Ghisellini et al., 2018; Klewe et al., 2022; López Ruiz et al., 2020; Mercader-Moyano et al., 2022; Mohammadiziaz and Bilec, 2023; Papamichael et al., 2022; Purchase et al., 2022), a holistic circular economy model must include (i) policies and strategies regarding the industry; (ii) sustainable by design waste management practices (including waste prevention, deconstruction, collection and segregation practices, on-sire sorting, efficient distribution of resources, transportation, etc.); (iii) implementation of R strategies of circular economy like repair (return used products to working conditions), refurbish (improve quality of used products), remanufacture (implementation of specific standards for remanufacturing used products), recycling (use of recycled or recyclable materials), reuse (CDW reuse where applicable) and material recovery (recover of materials or energy) for end-of-life management and (iv) monitoring of CDW accumulation through key metrics and tools (i.e. KPIs, life cycle assessment (LCA) models, material flow analysis, etc.) in order to create sufficient repositories for use (Figure 3). LCA is a methodology that evaluates the environmental impacts of a product or process throughout its entire life cycle from raw material extraction to end-of-life disposal. The construction industry is a significant contributor to global waste generation, with CDW accounting for a significant portion of the world’s waste stream. In this context, LCA can be a valuable tool for assessing the environmental impacts of construction waste and identifying opportunities for waste reduction and management. LCA can be used to assess the environmental impacts of construction waste in several ways. Firstly, LCA can be used to quantify the environmental impacts of construction waste disposal methods such as landfilling, incineration and recycling. By comparing these methods, LCA can help identify the most sustainable waste management strategies that minimize environmental impacts. For example, recycling of construction waste materials can have lower environmental impacts than landfilling or incineration. Secondly, LCA can be used to evaluate the environmental impacts of construction materials throughout their life cycle, including their production, use and disposal. This approach can help identify opportunities for waste reduction through design, material selection and construction practices. For example, using recycled materials in construction can reduce the environmental impacts of material production and waste generation. Thirdly, LCA can be used to assess the environmental impacts of construction projects as a whole. This approach considers the impacts of the entire construction process, including material selection, transportation, construction activities and waste generation. By evaluating the entire project, LCA can identify opportunities to minimize waste generation and environmental impacts (Jiang et al., 2023; Mesa et al., 2021; Stylianou et al., 2023).

### Monitoring of CDW in the framework of circular economy

In order to assess the level of achievement of the circular economy model related with CDW, the scientific community has proposed the adoption of different methods and tools such as the development of KPIs, digitalization (i.e. LCA, etc.), the use of big data approach and others (Jiang et al., 2023; Niyommaneerat et al., 2023). KPIs are computational sets and metrics that can be used to measure the effectiveness of CDW management practices. These KPIs can help track progress towards waste reduction targets, identify areas for improvement and evaluate the success of CDW management initiatives. Such KPIs include waste generation rate, waste disposal cost, material recovery rate, greenhouse gas emissions, waste accumulation rate, waste recycling rate and others. Such KPIs have been used in waste management research by various researchers, regardless of the classification and type of waste in question (Loizia et al., 2021; Lu et al., 2015; Papamichael et al., 2023).

González et al. (2021) proposed an innovative method for determining the degree of circularity for CDW, which quantifies the ratio of circular flows in three components: energy, water and raw material consumption and integrates them with the metric of social value added and economic benefit of the entire process throughout its life cycle, irrespectively of whether it is a new construction or a massive renovation work. As a result, five distinct metrics have been developed: the Water Circularity Index, the Energy Circularity Index, the Materials Circularity Index, the Social Circularity Index and Life Cycle Costing. The importance of KPIs was also mention by Fagone et al. (2023), proposing a set of KPIs for monitoring circularity processes related to five aspects including indicators for: (i) site quality (i.e. previously occupied land, contaminated land); (ii) material and resource (i.e. reuse of the existing structures, recovered/recycled materials, material from renewable sources, local material, disassembly material, certified material); (iii) water (i.e. percentage of water circularity); (iv) energy (i.e. percentage of renewable energy used) and (v) waste (i.e. diversion, reduction in total waste material, construction waste reduction, diversion of resources from landfill). With the same mindset, Taboada et al. (2020) proposed 15 new indicators for assessing efficiency and sustainability CDW management. KPIs were also introduced in a study conducted by Núñez-Cachot et al. (2020), in which the authors developed and tested a circular economy dashboard applicable to the construction sector and introduced a set of valid KPIs to assist in the management of a construction industry by visualizing its success in the circular economy. Besides that, Lu et al. (2015) stated that the waste generation rate (WGR) is commonly used as a KPI to benchmark CDW management performance in order to continuously improve performance. Using an available big dataset on CDW, they establish a set of more credible KPIs/WGRs in their study. Big data enables a comprehensive examination of all project categories over a relatively long period of time. It significantly reduces the randomness of sampling that has been observed in previous empirical studies of this type.

It is imperative to focus on enhancing the entire life cycle of CDW, starting from the design stage and extending to the deconstruction phase. This can include considerations such as conducting an LCA, utilizing prefabricated and modular construction techniques and incorporating elements that can be easily disassembled. Furthermore, it is important to address the end-of-life phase by emphasizing the reuse and recycling of building materials, as well as finding ways to eliminate composite materials that cannot be easily segregated. Alongside these efforts, the stage of use should also be emphasized, with a focus on maintenance, partial or total replacement and rehabilitation (Bertino et al., 2021).

CDW management should not only focus on the end-of-pipe solutions such as reuse and recycling but also on improving the ends of the cycle. This includes various stages starting from the design stage to the deconstruction stage. In the design stage, it is essential to consider the principles of the circular economy by incorporating the principles of the reduce, reuse and recycle hierarchy in the design process (Bertino et al., 2021). Additionally, LCA should be considered to minimize the environmental impact of the building throughout its entire life cycle (Stylianou et al., 2023). Moreover, prefabricated construction and modular design can improve the efficiency of the construction process, reduce waste generation and make it easier to disassemble and recycle the building elements at the end of their life. Disassembly of the elements of a building is a critical step to enable the reuse and recycling of materials. Furthermore, composite materials that cannot be segregated at the end of their life should be eliminated from the construction process, as they create a significant challenge for recycling. Instead, it is necessary to use materials that can be segregated and recycled or reused easily. The stage of use is another critical aspect of the CDW management strategy. Maintenance, partial or total replacement and rehabilitation of buildings should be done in a way that maximizes the lifespan of the building and its components. This can help reduce the need for demolition and disposal of materials and reduce the environmental impact associated with new construction. Improving the ends of the cycle and emphasizing the stage of use can contribute significantly to the reduction of CDW generation and the implementation of circular economy principles in the construction industry (Koda and Podlasek, 2023; Oluleye et al., 2023). By addressing all these aspects of the CDW cycle, we can achieve a more sustainable and circular approach to building construction and management, leading to significant benefits for the environment and society as a whole.

## Conclusion

Current frameworks on circular economy and CDW focus on promoting recycling and reusing in order to reduce the massive production of CDW, which poses a significant challenge to the environment and sustainability. These frameworks serve as guidelines for future studies and encourage the development of effective circular economy models that replace the linear approach to the design, construction, demolition and disposal of CDW. A circular model that enables materials to be recycled, reused or remanufactured, thereby extending their life cycle, is essential for achieving smooth transitions towards circularity while accounting for environmental factors, such as waste generation, societal factors, such as job opportunities, and economic factors, such as revenue from circularity activities. The scientific community’s future steps involve constructing appropriate schemes, frameworks and practices for managing CDW with considerations for risks, barriers and opportunities for circularity while using existing monitoring tools, such as KPIs, to evaluate the sector better.
